# The Impact of Nonsteroidal Anti‐Inflammatory Drugs on Radiographic Spinal Progression in Patients With Axial Spondyloarthritis: 10‐Year Results From an Inception Cohort

**DOI:** 10.1002/art.43447

**Published:** 2026-01-26

**Authors:** Murat Torgutalp, Valeria Rios Rodriguez, Fabian Proft, Mikhail Protopopov, Judith Rademacher, Hildrun Haibel, Joachim Sieper, Martin Rudwaleit, Denis Poddubnyy

**Affiliations:** ^1^ Department of Gastroenterology, Infectiology and Rheumatology (including Nutrition Medicine), Charité – Universitätsmedizin Berlin, Freie Universität Berlin and Humboldt‐Universität zu Berlin Berlin Germany; ^2^ Department of Rheumatology and Clinical Immunology, Charité – Universitätsmedizin Berlin Freie Universität Berlin and Humboldt‐Universität zu Berlin Berlin Germany; ^3^ Fraunhofer Institute for Translational Medicine and Pharmacology Berlin Germany; ^4^ Institute of Public Health, Charité – Universitätsmedizin Berlin Berlin Germany; ^5^ Berlin Institute of Health, Charité – Universitätsmedizin Berlin Berlin Germany; ^6^ Department of Internal Medicine and Rheumatology Klinikum Bielefeld Rosenhöhe Bielefeld Germany; ^7^ Division of Rheumatology, University of Toronto and Schroeder Arthritis Institute University Health Network Toronto Ontario Canada

## Abstract

**Objective:**

This study aims to investigate the impact of nonsteroidal anti‐inflammatory drug (NSAID) intake on radiographic spinal progression in axial spondyloarthritis (axSpA), considering different NSAID types (COX‐2 inhibitors [COX2i] and nonselective NSAIDs [ns‐NSAIDs]) and disease subgroups (radiographic [r‐axSpA] and nonradiographic [nr‐axSpA]).

**Methods:**

Leveraging data from the German Spondyloarthritis Inception Cohort (GESPIC), we conducted analyses on 252 patients with axSpA (139 with nr‐axSpA and 113 with r‐axSpA), who had minimum two sets of spinal radiographs. The outcome was progression in modified Stoke Ankylosing Spondylitis Spine Score (mSASSS) in two‐year intervals. We fitted sequential conditional mean models by using generalized estimating equations and adjusting for longitudinal repeated measures of exposure and time‐dependent confounders. We report β‐coefficients with 95% confidence intervals (CIs) for outcomes reflecting the progression in mSASSS per 10‐point increase in NSAID intake score.

**Results:**

At baseline, 201 (80.0%) patients were under NSAID treatment, with 46 (18%) taking COX2i and 156 (62%) taking ns‐NSAIDs, and mean total NSAID intake score was 38.3 ± 35.5. A 10‐point increase in NSAID intake score was associated with retardation of radiographic progression (β = −0.052, 95% CI: −0.097 to −0.007), with this effect being most pronounced in patients with r‐axSpA (β = −0.077, 95% CI: −0.152 to −0.003). COX2i showed a slightly lower point estimate (although not statistically significant) in progression compared to ns‐NSAIDs among all patients with axSpA (β = −0.061 and −0.045, respectively).

**Conclusion:**

Our findings suggest a beneficial effect of higher NSAID intake, particularly COX2i, on slowing radiographic progression in axSpA. These findings may help inform therapeutic strategies, particularly in r‐axSpA, although further research is needed for nr‐axSpA.

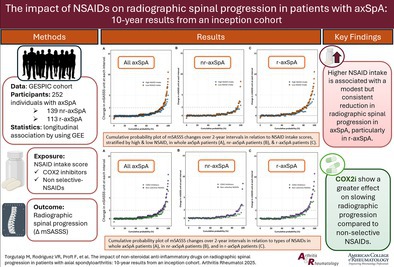

## Graphical abstract

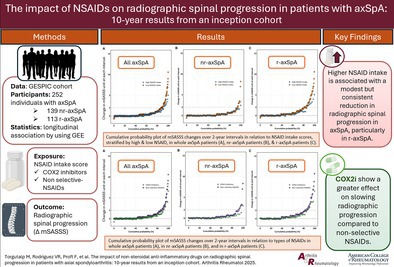

## INTRODUCTION

Axial spondyloarthritis (axSpA) is a chronic inflammatory disease that primarily affects the axial skeleton, including the sacroiliac joints and spine.^1^ This inflammation causes pain, stiffness, and structural damage in the affected joints. If left uncontrolled, inflammation may lead to radiographic progression in the spine characterized by new bone formation, which significantly contributes to functional limitations and impaired mobility.^2^, ^3^

Over the past two decades, the pharmaceutical treatment of axSpA has expanded significantly with the introduction of biologic therapies targeting tumor necrosis factor (TNF), interleukin‐17, and JAK.^4^ Despite these advancements, current guidelines recommend nonsteroidal anti‐inflammatory drugs (NSAIDs) as the initial therapy for the management of axSpA symptoms.^5^, ^6^ Beyond symptomatic pain relief, NSAIDs have also been investigated for their disease‐modifying impacts on the course of axSpA, particularly with regard to radiographic spinal progression. The potential disease‐modifying effects of NSAIDs are attributed to the inhibition of cyclooxygenase (COX) enzymes, leading to reduced production of prostaglandins that play a key role in inflammation and osteoblastogenesis.^7^, ^8^

Despite these potential benefits, existing literature on the impact of NSAIDs on radiographic spinal progression in axSpA presents conflicting findings.^9^, ^10^, ^11^, ^12^, ^13^ This inconsistency is primarily due to variations in the types of NSAIDs used, the populations studied, and the study designs. Although some research suggests that NSAIDs, particularly COX2 inhibitors (COX2i), may reduce structural damage,^9^, ^10^ other studies, like the ENRADAS and CONSUL trials, present a more cautious view or have not found such a clear benefit.^12^, ^13^ Moreover, the majority of these studies have targeted patients with established radiographic disease (radiographic axSpA [r‐axSpA], previously referred to as ankylosing spondylitis), with only one cohort study involving patients with nonradiographic axSpA (nr‐axSpA).^11^

Given the mixed results in previous studies, the present study aims to investigate the association between NSAID intake and radiographic spinal progression in patients with axSpA. The secondary objectives include assessing the differential impacts of NSAID types (COX2i and nonselective NSAIDs [ns‐NSAIDs]) and examining whether this relationship varies among disease subgroups categorized by their classification status (r‐axSpA and nr‐axSpA).

## METHODS

### Cohort design and participants

We performed analyses on the data from patients enrolled in German Spondyloarthritis Inception Cohort (GESPIC), an ongoing longitudinal study designed to investigate clinical and radiographic outcomes of patients with axSpA. The study design, methodology, and eligibility criteria were documented in detail in previous publications.^14^, ^15^ In summary, inclusion criteria required patients to have either r‐axSpA meeting the modified New York criteria with a symptom duration of less than 10 years or nr‐axSpA meeting the European Spondyloarthropathy Study Group criteria (with a slight modification) with a symptom duration of less than five years. The r‐axSpA or nr‐axSpA classification was based on central reading of baseline sacroiliac radiographs as described previously; when central reading results were not available, the local rheumatologist's assessment served as the basis for classification.^15^, ^16^ Due to observational nature of the cohort, there were no treatment restrictions, allowing physicians to prescribe treatments based on their clinical judgment. Data on demographics, disease‐relevant characteristics (such as activity, function, mobility, and presence of extra musculoskeletal manifestations), laboratory (C‐reactive protein [CRP], and HLA‐B27), and treatments (NSAIDs, TNF inhibitors [TNFi]) were systematically collected at baseline, every six months for the first two years, and then annually through year 10. Disease activity was assessed by using the Bath Ankylosing Spondylitis Disease Activity Index, CRP levels, and patient global assessment of disease activity. The Axial Spondyloarthritis Disease Activity Score (ASDAS) was also calculated.^17^, ^18^

The study protocol was approved by the ethics committee of the coordinating center (Charité—Universitätsmedizin Berlin, Germany/ethics approval number 188‐19) and by local ethical committees of participating centers. Participants gave informed consent to participate in the study before taking part. Patients and/or the public were not involved in the design, conduct, reporting, or dissemination plans of this research. The data that support the findings of this study are available on request from the corresponding author. The data are not publicly available due to privacy or ethical restrictions.

### Exposure definition: NSAID types and NSAID intake score calculation

During the 10‐year follow‐up, data on NSAID types, their daily dosage, frequency of intake, and any side effect of NSAIDs were recorded at each visit. The most used COX2i were celecoxib and etoricoxib, whereas diclofenac, ibuprofen, naproxen, and indomethacin were the most used ns‐NSAIDs.

We assessed NSAID intake using the score previously proposed by the Assessment of Spondyloarthritis International Society, which has been designed for the quantification of NSAID intake.^19^ This score primarily incorporates two key elements: daily dose of NSAID, determined based on the equivalent dose conversion for each NSAID, and duration of NSAID consumption over the relevant time period. Scores for NSAID intake range from 0 to 100, with 0 representing no NSAID use and 100 representing of a full dose NSAID use. For the current analysis, we calculated the total NSAID intake score at each visit for each type and averaged these values within each two‐year radiographic interval. This approach allowed us to capture longitudinal exposure, including any discontinuation or switching of NSAIDs during follow‐up. We used the total NSAID intake score as the primary exposure of interest for the main analyses. For analyses of NSAID subtypes, we classified patients according to the type of NSAID (COX2i or ns‐NSAID) that they used for at least one year within the interval. To help readers better understand and interpret the findings, we reported the results as a 10‐point increase in the total NSAID intake score. We also assessed the total causal effect estimate for each type of NSAIDs to explore their associations with radiographic progression (details are provided in the Statistical analysis section).

### Outcome definition: scoring of spinal radiographs and outcomes

Lateral cervical and lumbar spinal radiographs were obtained at baseline and every two years thereafter with a maximum six time points (baseline and years 2, 4, 6, 8, and 10), as specified in the protocol. Patients with at least two consecutive sets of cervical and lumbar spinal radiographs were eligible for analysis, resulting in a final sample of 252 participants.

Three trained and calibrated readers evaluated spine radiographs by using the modified Stoke Ankylosing Spondylitis Spine Score (mSASSS).^20^ Readers were blinded to all clinical data except for the chronological order of the images. A total mSASSS, ranging from 0 to 72, was calculated for each reader. In cases in which radiographic data were missing at a given time point, we used the following imputation method: (1) if the mSASSS was missing at a time point, and if the scores were identical for the previous and subsequent time points, the missing score was imputed with the available value; (2) for individual missing vertebral corners, a score of 3 (indicating a total ankylosis) at an earlier time point was used to impute the missing score at a later time point; and similarly, but in reverse, a score of 0 (indicating no abnormality) at a later time point was used to impute the missing score at an earlier time point.

We made no further imputations and calculated the final mSASSS for each patient and time point as the mean scores of three readers. We assessed the reliability of the assessments by the intraclass correlation coefficient for status and change scores of mSASSS among readers. Our outcome was the progression in mSASSS in a two‐year interval, which was determined as a difference in final mSASSS between the two time points, modeled as a dependent variable.

### Covariates

We used directed acyclic graphs (DAGs) to formalize causal structures and determine the total effect of NSAID intake on radiographic spinal progression.^21^ Supplementary Figure 1 represents the DAG, illustrating the exposure (a 10‐point increase in the total NSAID intake score over a two‐year interval) and outcome (progression in mSASSS); furthermore, this figure denotes the temporal sequence of variables by “t − 1” and “t” for previous and current time points, respectively. We identified covariates from the published literature and expert knowledge to define the exposure‐outcome relationship conceptually. These covariates included status and time‐averaged variables. Status variables, measured at the beginning of each interval, were sex, age, symptom duration, smoking status, classification status (classification as r‐axSpA or nr‐axSpA), HLA‐B27 positivity, body mass index, and mSASSS; whereas time‐averaged variables, calculated over the two‐year interval, were ASDAS, side effects due to NSAID intake, conventional disease‐modifying antirheumatic drugs (csDMARD) use, and TNFi exposure. Although data regarding inflammation on magnetic resonance imaging (MRI), history of cardiovascular disease, and other potential contributors of mechanical pain (eg, degenerative disc disease, osteoarthritis) were not collected in the cohort's case report form, we nonetheless included them into our DAG because of their potential causal relationships with exposure or outcome.

### Statistical analyses

For the descriptive data, we used mean ± SD and number (percentage) for numerical and categorical variables, respectively. The statistical model structure we used is as follows: We used a per‐10‐point increase in the mean NSAID intake score over the two‐year interval as the main exposure variable (independent variable of interest). The β‐coefficients for the primary outcome reflected the progression in mSASSS per 10‐point increase in NSAID intake.

We analyzed longitudinal association between NSAID intake and radiographic spinal progression using generalized estimating equations (GEEs) with an independent working correlation structure. Given the design of the study, which included longitudinal repeated measures of exposure and outcome and time‐dependent confounders, we used sequential conditional mean models (SCMMs) to minimize potential GEE bias as described by Keogh et al.^22^ Briefly this approach addresses biases that may arise in traditional regression analyses due to time‐varying variables affected by prior exposures or covariates and can be incorporated with propensity score (PS) adjustment.

We built three different models including adjustments for both prior covariates (ASDAS or TNFi exposure) and prior exposure (total NSAID intake score) and implemented SCMMs to determine the total causal effect of NSAID intake on radiographic spinal progression. To explore the robustness of our findings, we conducted sensitivity analyses incorporating PS adjustments within the SCMMs. The PS represents the estimated probability of an individual receiving the exposure at a specific time point (“t”), given their prior and current characteristics. For our continuous exposure variable, we fitted a linear regression model to estimate PS across all time points combined. This model accounted for the influence of relevant variables on exposure or outcome. Subsequently, the estimated PS was integrated into the SCMM for further sensitivity analysis. This step allowed us to control potential confounding factors that might have biased the initial estimates of the association between NSAID intake and radiographic progression. We also tested for all relevant interactions between mSASSS and NSAID exposure, and other covariates.

To assess the effect of two NSAIDs types over two‐year radiographic intervals, we also assessed the following two variables as exposure variables: COXi intake score and ns‐NSAID intake score, per 10‐point increase for both variables. Considering that our main exposure variable was the total NSAID intake score, a compositional (also described as comparative) data set, which consists of these two distinct components, we, therefore, used all‐component models to calculate the effect of each NSAID types on radiographic spinal progression. Briefly, this model targets the total causal effects of two NSAID types separately and does not include the total NSAID intake score (main exposure variable), and, therefore, does not allow opening a closed path by a collider. The estimated coefficient is expected to provide an unbiased total causal effect estimate of the individual NSAID types as described by Tomova et al.^23^

We used DAGitty version 3.1, a web application, to formalize causal structures.^21^ For main statistical analyses, we used the “*geepack*” package in R software version 4.3.3 (R Project for Statistical Computing).^24^, ^25^ We calculated and reported parameter estimates (β) with 95% confidence intervals (CIs).

## RESULTS

### Baseline characteristics and NSAID intake

The GESPIC core cohort enrolled 525 patients with axSpA. For our analysis, we focused on 252 patients who had at least two consecutive radiographs (113 r‐axSpA and 139 nr‐axSpA). Table 1 presents a comparison of the baseline characteristics of included and excluded patients.

**Table 1:** Baseline characteristics of included and excluded patients*

Variable	Overall (N = 525)	Included (n = 252)	Excluded (n = 273)
Age at baseline, y	35.7 ± 10.3	35.9 ± 10.2	35.4 ± 10.4
Male sex, n (%)	286 (54)	125 (50)	161 (59)
Symptom duration, y	3.9 ± 2.7	3.9 ± 2.5	4.0 ± 2.9
Family history of SpA, n (%)	158 (30)	86 (34)	72 (26)
HLA‐B27 positivity, n (%)	406 (78)	199 (80)	207 (76)
Ever smoked, n (%)	132 (25)	68 (27)	64 (23)
BMI at baseline, kg/m^2^	24.9 ± 4.8	24.7 ± 4.8	25.1 ± 4.7
Ever uveitis, n (%)	86 (16)	46 (18)	40 (15)
Ever psoriasis, n (%)	53 (10)	29 (12)	24 (9)
Ever IBD, n (%)	14 (3)	7 (3)	7 (3)
CRP, mg/L	10.7 ± 17.3	8.8 ± 14.2	12.4 ± 19.6
ASDAS	2.6 ± 1.0	2.5 ± 1.0	2.7 ± 1.0
BASDAI (0–10 points NRS)	3.9 ± 2.1	3.8 ± 2.1	4.1 ± 2.0
BASFI (0–10 points NRS)	2.8 ± 2.4	2.7 ± 2.3	2.8 ± 2.4
BASMI (0–10 points NRS)	1.5 ± 1.6	1.6 ± 1.6	1.4 ± 1.7
Use of systemic glucocorticoids, n (%)	48 (9)	17 (7)	31 (11)
Use of csDMARDs, n (%)	132 (25)	68 (27)	64 (23)
Use of TNFi, n (%)	13 (2)	8 (3)	5 (2)
Use of NSAID, n (%)			
No NSAID	111 (21)	50 (20)	61 (22)
COX2i	103 (20)	46 (18)	57 (21)
ns‐NSAID	311 (59)	156 (62)	155 (57)
Total NSAID intake score	38.3 ± 35.5	37.1 ± 33.8	39.4 ± 37.1
Mean COX2i intake score	9.1 ± 22.8	7.7 ± 19.3	10.4 ± 25.5
Mean ns‐NSAID intake score	29.2 ± 35.1	29.4 ± 34.4	29.0 ± 35.8
Presence of syndesmophytes, n (%)	66 (16)	40 (16)	26 (16)
mSASSS, units	2.5 ± 5.9	2.5 ± 6.4	2.7 ± 4.9
Patients with r‐axSpA, n (%)	248 (47)	113 (45)	135 (49)

* Values described as mean ± SD unless otherwise indicated. ASDAS, Axial Spondyloarthritis Disease Activity Score; BASDAI, Bath Ankylosing Spondylitis Disease Activity Index; BASFI, Bath Ankylosing Spondylitis Functional Index; BASMI, Bath Ankylosing Spondylitis Metrology Index; BMI, body mass index; COX2i, selective cyclooxygenase‐2 inhibitor; CRP, C‐reactive protein; csDMARD, conventional synthetic disease‐modifying antirheumatic drug; IBD, inflammatory bowel disease; mSASSS, modified Stroke Ankylosing Spondylitis Spine Score; NRS, numeric rating scale; NSAID, nonsteroidal anti‐inflammatory drug; ns‐NSAID, nonselective NSAID; r‐axSpA, axSpA, axial spondyloarthritis; SpA, spondyloarthritis; TNFi, tumor necrosis factor alpha inhibitor.

The included and excluded groups had similar characteristics, with a mean age of 35.7 ± 10.3 years and a mean symptom duration of 3.9 ± 2.7 years. However, included patients were less frequently male (50% vs 59%) and had a lower mean ASDAS (2.5 vs 2.7) than excluded patients. Additionally, included patients had slightly lower mean baseline mSASSS (2.6 vs 2.7). However, there was no difference in the proportion of patients with syndesmophytes at baseline or the proportion of patients with r‐axSpA. Only 8 (3%) of the included patients were treated with a TNFi, whereas 68 (27%) were receiving a csDMARD.

At baseline, 201 (80.0%) of the included patients were receiving NSAID treatment, with 46 (18%) taking COX2i and 156 (62%) taking ns‐NSAIDs. The mean total NSAID intake score was 38.3 ± 35.5, with scores of 9.1 ± 22.8 and 29.2 ± 35.1 for COX2i and ns‐NSAIDs, respectively.

### The effect of total NSAID intake on radiographic spinal progression

Adjusted multivariable GEE analyses showed a consistent association between increased NSAID intake and reduced radiographic progression in patients with whole axSpA in three different models (Table 2). Model 1 demonstrated an inverse association between total NSAID intake score and progression in mSASSS, with a moderate reduction of progression per 10‐point NSAID intake (adjusted β = −0.052, 95% CI: −0.097 to −0.007), representing a borderline association. Models 2 and 3, which included different adjustment sets, yielded similar results, with a β of −0.051 for both. Similarly, including PS adjustments in the analyses further strengthened the observed association and provided comparable results (β's were −0.056 for all models with PS in Table 2).

**Table 2:** Effect of per 10‐point increase in total NSAID intake score on progression in mSASSS*

Model^a^	Exposure^b^	All axSpA^c^		nr‐axSpA		r‐axSpA	
		Adjusted β	95% CI	Adjusted β	95% CI	Adjusted β	95% CI
Model 1^d^	Total NSAID intake score	−0.052	−0.097 to −0.007	−0.014	−0.042 to 0.014	−0.077	−0.152 to −0.003
Model 1 with PS	Total NSAID intake score	−0.056	−0.101 to −0.010	−0.012	−0.041 to 0.017	−0.089	−0.164 to −0.014
Model 2^e^	Total NSAID intake score	−0.051	−0.097 to −0.006	−0.007	−0.035 to 0.021	−0.081	−0.157 to −0.005
Model 2 with PS	Total NSAID intake score	−0.056	−0.102 to −0.009	−0.009	−0.037 to 0.020	−0.092	−0.169 to −0.014
Model 3^f^	Total NSAID intake score	−0.051	−0.096 to −0.006	−0.007	−0.032 to 0.019	−0.075	−0.150 to −0.000
Model 3 with PS	Total NSAID intake score	−0.056	−0.103 to −0.009	−0.011	−0.037 to 0.015	−0.088	−0.165 to −0.012

* ASDAS, Axial Spondyloarthritis Disease Activity Score; axSpA, axial spondyloarthritis; CI, confidence interval; EMM, extra musculoskeletal manifestation; mSASSS, modified Stroke Ankylosing Spondylitis Spine Score; nr‐axSpA, nonradiographic axSpA; NSAID, nonsteroidal anti‐inflammatory drugs; PS, propensity score; r‐axSpA, radiographic axSpA; TNFi, tumor necrosis factor alpha inhibitor

^a^ Parameter estimates from all the multivariable models adjusted for sex, presence of EMMs, time‐averaged ASDAS in the current interval, mSASSS at the beginning of the interval, and total NSAID intake score in the previous interval.

^b^ Reference is per 10‐point increase in NSAID intake score.

^c^ Models in all axSpA groups also include classification as radiographic axSpA at the beginning of each interval in the adjustment set.

^d^ Model 1 also adjusted for age at the beginning of the interval, smoking in the interval, and time‐averaged ASDAS in the previous interval.

^e^ Model 2 also adjusted for age at the beginning of the interval and TNFi exposure (≥12 months) in the previous interval.

^f^ Model 3 also adjusted for symptom duration at the beginning of the interval and TNFi exposure (≥12 months) in the previous interval.

Upon stratifying patients according to their classification status, the analyses revealed a trend toward a greater benefit of NSAID intake on radiographic progression between nr‐axSpA and r‐axSpA subsets. Specifically, we observed a numerically larger reduction in radiographic progression per 10‐point increase in NSAID intake score in the r‐axSpA group, with an adjusted β of −0.077 (95% CI: −0.152 to −0.003) in model 1 (Table 2). However, the effect was less pronounced in the nr‐axSpA population, with a β of −0.014 (95% CI: −0.042 to 0.014). The results indicate that NSAID intake has a more significant impact on reducing radiographic progression in patients with r‐axSpA than in those with nr‐axSpA. This observed trend was consistent also in other models (βs were −0.081 and −0.075, for model 2 and 3, respectively) and PS adjusted models (βs were −0.092 and −0.088, for model 2 and 3, respectively), as shown in Table 2.

**Figure 1:**
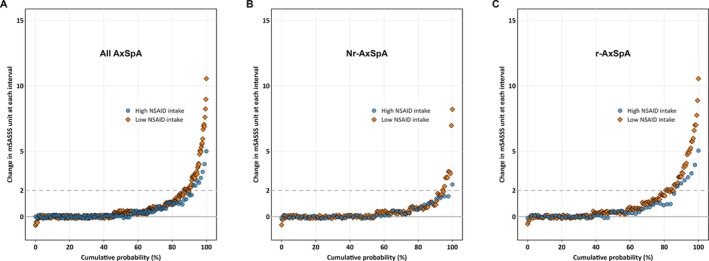
Cumulative probability plot of mSASSS changes over two‐year intervals in relation to NSAID intake scores, stratified by high and low NSAID, in (A) patients with whole axSpA, (B) patients with nr‐axSpA, (C) and patients with r‐axSpA. axSpA, axial spondyloarthritis; mSASSS, modified Stoke Ankylosing Spondylitis Spinal Score; nr‐axSpA, nonradiographic axSpA; NSAID, nonsteroidal anti‐inflammatory drug; r‐axSpA, radiographic axSpA.

Figure 1 presents the cumulative probability plot, highlighting the divergence in mSASSS change at individual interval level between high (NSAID intake score ≥50) and low (NSAID intake score <50) NSAID intake groups. This trend appeared numerically stronger in the r‐axSpA subgroup.

### The effect of different NSAID types on radiographic spinal progression

Table 3 displays the effects of NSAID types on radiographic spinal progression. Our multivariable analyses suggested numerically larger reduction in progression with COX2i compared to ns‐NSAIDs in all patients with axSpA. Model 1 showed a reduction of 0.061 points in mSASSS progression for each 10‐point increase in COX2i use (β: −0.061, 95% CI: −0.138 to 0.015), compared with a reduction of 0.045 points for the same increase in ns‐NSAID use (β: −0.045, 95% CI: −0.089 to −0.001). Although point estimates suggested a stronger effect for COX2i, the CIs overlapped, and the difference was not statistically significant. Consistent with main analyses, models 2 and 3, and all models incorporating PS adjustments to evaluate the total effect of NSAID types, showed similar trends, with numerically larger reductions in radiographic progression associated with COX2i compared to ns‐NSAIDs in the whole axSpA population (Table 3).

**Table 3:** Effect of per 10‐point increase in subtypes of NSAIDs (COX2i and ns‐NSAID intake Score) on Progression in mSASSS*

Model^a^	Exposure^b^	All axSpA^c^		nr‐axSpA		r‐axSpA	
		Adjusted β	95% CI	Adjusted β	95% CI	Adjusted β	95% CI
Model 1^d^	COX2i intake score	−0.061	−0.138 to 0.015	−0.031	−0.064 to 0.003	−0.073	−0.191 to 0.045
Model 1^d^	ns‐NSAID intake score	−0.045	−0.089 to −0.001	−0.010	−0.043 to 0.023	−0.076	−0.152 to −0.000
Model 1 with PS	COX2i intake score	−0.064	−0.141 to 0.012	−0.029	−0.061 to 0.004	−0.084	−0.203 to 0.035
Model 1 with PS	ns‐NSAID intake score	−0.048	−0.093 to −0.004	−0.008	−0.042 to 0.026	−0.088	−0.165 to −0.012
Model 2^e^	COX2i intake score	−0.062	−0.137 to 0.014	−0.020	−0.053 to 0.013	−0.078	−0.204 to 0.048
Model 2^e^	ns‐NSAID intake score	−0.044	−0.087 to −0.000	−0.004	−0.038 to 0.030	−0.078	−0.152 to −0.004
Model 2 with PS	COX2i intake score	−0.069	−0.148 to 0.011	−0.028	−0.061 to 0.005	−0.090	−0.217 to 0.037
Model 2 with PS	ns‐NSAID intake score	−0.047	−0.091 to −0.002	−0.004	−0.038 to 0.029	−0.088	−0.164 to −0.012
Model 3^f^	COX2i intake score	−0.061	−0.137 to 0.015	−0.018	−0.049 to 0.013	−0.075	−0.203 to 0.054
Model 3^f^	ns‐NSAID intake score	−0.044	−0.087 to −0.001	−0.005	−0.037 to 0.026	−0.071	−0.141 to −0.001
Model 3 with PS	COX2i intake score	−0.069	−0.149 to 0.012	−0.028	−0.060 to 0.003	−0.090	−0.220 to 0.041
Model 3 with PS	ns‐NSAID intake score	−0.047	−0.092 to −0.002	−0.008	−0.038 to 0.023	−0.083	−0.156 to −0.011

* ASDAS, Axial Spondyloarthritis Disease Activity Score; axSpA, axial spondyloarthritis; CI, confidence interval; COX2i, selective cyclooxygenase‐2 inhibitor; EMM, extra musculoskeletal manifestation; mSASSS, modified Stroke Ankylosing Spondylitis Spine Score; nr‐axSpA, nonradiographic axSpA; NSAID, nonsteroidal anti‐inflammatory drug; ns‐NSAID, nonselective NSAID; PS, propensity score; r‐axSpA, radiographic axSpA; TNFi, tumor necrosis factor alpha inhibitor.

^a^ Parameter estimates from all the multivariable models adjusted for sex, presence of EMMs, time‐averaged ASDAS in the current interval, mSASSS at the beginning of the interval, and total NSAID intake score in the previous interval.

^b^ Models in all axSpA groups also include classification as radiographic axSpA at the beginning of each interval in the adjustment set.

^c^ Reference is per 10‐point increase in corresponding intake score for each exposure. All models include per 10‐point increase in COX2i intake score and ns‐NSAID intake score (these models did not adjust for total NSAID intake score).

^d^ Model 1 also adjusted for age at the beginning of the interval, smoking in the interval, and time‐averaged ASDAS in the previous interval.

^e^ Model 2 also adjusted for age at the beginning of the interval and TNFi exposure (≥12 months) in the previous interval.

^f^ Model 3 also adjusted for symptom duration at the beginning of the interval and TNF exposure (≥12 months) in the previous interval.

**Figure 2:**
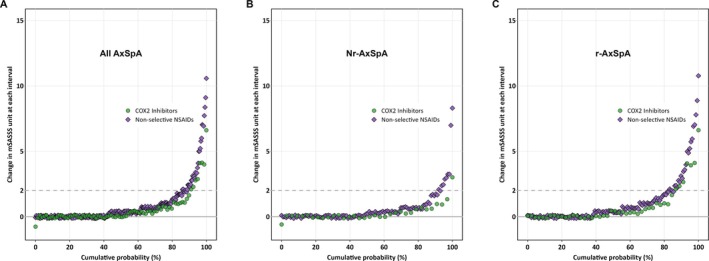
Cumulative probability plot of mSASSS changes over two‐year intervals in relation to types of NSAIDs in (A) patients with whole axSpA, (B) patients with nr‐axSpA, (C) and patients with r‐axSpA. axSpA, axial spondyloarthritis; COX2, selective cyclooxygenase‐2; mSASSS, modified Stoke Ankylosing Spondylitis Spinal Score; nr‐axSpA, nonradiographic axSpA; NSAID, nonsteroidal anti‐inflammatory drug; r‐axSpA, radiographic axSpA. Color figure can be viewed in the online issue, which is available at http://onlinelibrary.wiley.com/doi/10.1002/art.43447/abstract.

After stratification by classification status, we extended our analysis to differentiate between COX2i and ns‐NSAIDs. We performed multivariable analyses using the same covariate adjustments as in the models for the whole axSpA group and found similar reductions in radiographic spinal progression in the r‐axSpA subgroup for both types of NSAIDs (Table 3). In patients with r‐axSpA, each 10‐point increase in COX2i intake score was associated with a 0.073‐point reduction in mSASSS progression (β = −0.073, 95% CI: −0.191 to 0.045). Notably, a 10‐point increase in ns‐NSAIDs also corresponded to a similar reduction in mSASSS progression (β = −0.076, 95% CI: −0.152 to 0.000) in the r‐axSpA group. However, like our analyses for total NSAID intake score, we observed no statistically significant association for either COX2i or ns‐NSAID use in the nr‐axSpA group in the nr‐axSpA group (β: −0.031, 95% CI: −0.064 to 0.003 and β = −0.010, 95% CI: −0.043 to 0.023, respectively, in model 1). These observed trends were consistent across all other models used in stratified analyses (Table 3). Figure 2 shows a cumulative probability plot that illustrates a trend toward the lower probability of mSASSS progression over two years for intervals covered by COX2i compared to intervals covered by an ns‐NSAID, at the level of all individual intervals.

## DISCUSSION

Our results consistently showed an association between higher NSAID intake and a retardation in radiographic spinal progression in axSpA across different models. Specifically, a 10‐point increase in the total NSAID intake score was associated with a moderate reduction in mSASSS progression (β ranged from −0.051 to −0.058). After stratification, this association was more evident in patients with established radiographic disease (r‐axSpA). Additionally, the analyses revealed a stronger effect of COX2i compared to ns‐NSAIDs on reducing radiographic spinal progression in whole axSpA population. We believe that these findings provide a robust estimation of the longitudinal impact of NSAIDs (selective COX2 and nonselective inhibitors) on radiographic spinal progression in axSpA (in both radiographic and nonradiographic forms).

The relationship between NSAID intake and radiographic spinal progression in axSpA remains a topic of considerable debate. Although some previous research supported the idea that NSAIDs may slow radiographic progression, the magnitude of this effect and its applicability to different types of NSAIDs varied.^9^, ^10^, ^11^, ^12^, ^13^ Similar to ours, some studies suggested that NSAIDs might have a beneficial effect in slowing structural damage. For instance, a 2005 randomized controlled trial (RCT) showed a reduction in radiographic spinal progression in patients with r‐axSpA who received continuous treatment of celecoxib, a COX2i, compared to those treated on demand.^9^ The effect of COX2i on slowing progression was more evident in continuous group, particularly in patients with high disease activity, indicating a potential disease‐modifying effect.^10^ The two‐year results of GESPIC confirmed this finding and demonstrated that higher NSAID intake had a protective effect on radiographic progression, especially in the r‐axSpA group, but the study did not differentiate between the effects of COX2i and ns‐NSAID intake.^11^ However, in 2015 the ENRADAS study showed no significant difference between continuous and on‐demand treatment with diclofenac, an ns‐NSAID, on structural progression in r‐axSpA patients.^12^ The most recent CONSUL study further complicated the narrative by questioning the added benefit of celecoxib in combination therapy with a TNFi.^13^ Although this study demonstrated a numerical effect of combination with celecoxib on radiographic progression compared to TNFi alone in patients with r‐axSpA with high risk for radiographic progression, this difference did not reach statistical significance. These discrepancies among studies can be explained by several factors, chief among which are methodologic differences between studies. Variations in results may be attributable to differences in study design (observational vs RCTs), sample size, type of NSAID used, dosage, and duration of treatment. The observed differences may also be attributed to the specific population studied. It is important to note that all RCTs discussed above included only patients with r‐axSpA, and only the cohort study included the entire axSpA population.

These studies highlighted the complexity of understanding the impact of NSAIDs on structural progression in axSpA, although there is some evidence supporting their use in reducing radiographic progression, especially in patients with r‐axSpA or with COX2i. The results of the present study suggest a trend toward stronger effects of COX2i compared to ns‐NSAIDs in all patients with axSpA, which is consistent with previous research. Additionally, we found that ns‐NSAIDs also had a protective effect on progression in r‐axSpA group, in contrast to above‐mentioned ENRADAS study.^12^

This inverse association between NSAID intake and radiographic progression can be explained by several mechanisms. First, NSAIDs act primarily by inhibiting COX enzymes and exerting their anti‐inflammatory effects, leading to a reduction in the production of inflammatory mediators, mainly prostaglandins.^7^ As inflammation plays a key role in the development and progression of spinal damage in axSpA, NSAIDs may help to slow the process of damage by reducing inflammation. Second, NSAIDs effectively relieve pain and are therefore used for pain management, which is the main symptom of axSpA. Improved pain control may lead to increased physical activity and mobility, potentially reducing the risk of spinal stiffness and deformity, which may contribute to the slowing of damage. Third, although the specific mechanisms remain to be investigated, some studies suggest that COX2 selectivity may have greater anti‐inflammatory and potentially disease‐modifying effects compared to ns‐NSAIDs—beyond their pain‐relieving effects—and this may result in a relatively superior effect on structural damage.^8^, ^26^ Therefore, COX2i potentially offer a more targeted anti‐inflammatory effect that may effectively block pathways leading to bone fusion. Finally, we observed that the effect of both NSAIDs on structural damage appeared numerically stronger in the r‐axSpA group than in the nr‐axSpA group. We suggest that this effect may be attributable to already present structural damage (ie, destructive/erosive changes), which is more common in advanced disease, being more likely to lead to more radiographic progression, and that there may be an interference between the inhibitory effect of NSAIDs on new bone formation and radiographic progression in advanced disease.

These findings have implications for clinical practice in potentially slowing disease progression, particularly suggesting that the beneficial effect of COX2i and ns‐NSAIDs in reducing radiographic progression is more pronounced in patients with established radiographic disease (r‐axSpA). However, further investigations are required to determine the differences regarding the effect of NSAIDs based on disease classification. If confirmed, these findings could be considered in treatment strategies by tailoring the use of NSAIDs to specific patient subgroups based on disease characteristics and radiographic features. Although NSAIDs are currently recommended as the first‐line therapy to manage axSpA symptoms, their potential effect on slowing structural damage may add another dimension to their therapeutic value. Although the absolute effect sizes observed in our study were small, even modest reductions in radiographic progression may accumulate over time and translate into clinically meaningful differences, especially considering the irreversible nature of structural spinal damage in axSpA. At the population level, such reductions may also help decrease overall disease burden. However, it is important to determine the most appropriate type of NSAID, dosage, and duration of treatment to minimize potential side effects, such as gastrointestinal and cardiovascular risks, and maximize benefits.^27^ Beyond safety considerations, studies have shown that controlling inflammation with NSAIDs may improve survival, as higher disease activity in axSpA is associated with increased mortality.^28^, ^29^ This highlights the importance of carefully balancing the risks and potential long‐term benefits of NSAID use.

This study possesses several strengths, the foremost of which is the incorporation of the SCMMs. These models account for longitudinal repeated measures of exposure and outcome, as well as time‐dependent confounders. The design of our study takes advantage of this methodology to mitigate biases that often occur in traditional GEE regression analyses when time‐varying variables may be affected by past exposures or covariates. Evidence from previous research, including simulation studies, highlights the ability of SCMMs to accurately estimate beta coefficients for associations between time‐varying exposures and outcomes. Their effectiveness is comparable to that of marginal structural models, which use inverse probability treatment weighting of PSs to handle this complex study design.^22^ Second, our longitudinal statistical modeling isolates the within‐patient effect, providing clinicians with the best possible evidence when conducting an RCT is not always feasible. Moreover, incorporating PS adjustment enhances the reliability of our findings. The further strength of this study is its inclusion of entire axSpA population, including both r‐axSpA and nr‐axSpA, and its assessment of different types of NSAIDs together, unlike other studies that focus either on specific subgroups or on a specific type of NSAID. Our findings underscore the importance of investigating potential differences in treatment response across the whole axSpA spectrum and different types of NSAIDs. In addition, based on our current knowledge, this study highlights for the first time that NSAIDs may have a modest but consistent effect on structural damage even in nr‐axSpA population. Finally, usage of systematically collected data from a large, well‐defined cohort with extended follow‐up allowed a comprehensive and robust analysis.

However, it is important to acknowledge some limitations. First, the cohort's case report form did not provide data on certain factors that could influence the outcome, such as inflammation on MRI, or affect exposure, such as specific comorbidities or alternative causes of pain. Nonetheless, we considered all these factors in our DAG to describe causal relationships when building our models, and we included ASDAS (with CRP) in the multivariable analyses. Second, we excluded patients who did not have consecutive spinal radiographs required for radiographic progression calculation. Although the characteristics of included and excluded patients were similar, this exclusion may have introduced attrition bias. The third limitation concerns the collection of NSAID usage information, which relied on patient‐reported intake and may therefore be subject to recall bias. Finally, radiographic progression in nr‐axSpA was generally low, possibly independent of therapy. This likely reflects the lower level of baseline structural damage in nr‐axSpA, which is associated with less subsequent progression, and may explain the limited NSAID effect observed in this subgroup.

In conclusion, our study adds evidence to support the beneficial effect of higher NSAID intake on slowing radiographic spinal progression in patients with axSpA, and particularly in patients with r‐axSpA. More specifically, COX‐2i showed a slightly superior effect compared to ns‐NSAIDs across the whole axSpA spectrum.

## AUTHOR CONTRIBUTIONS

All authors contributed to at least one of the following manuscript preparation roles: conceptualization AND/OR methodology, software, investigation, formal analysis, data curation, visualization, and validation AND drafting or reviewing/editing the final draft. As corresponding author, Dr Torgutalp confirms that all authors have provided the final approval of the version to be published, and takes responsibility for the affirmations regarding article submission (eg, not under consideration by another journal), the integrity of the data presented, and the statements regarding compliance with institutional review board/Declaration of Helsinki requirements.

## ROLE OF THE STUDY SPONSOR

Abbott/AbbVie, Amgen, Centocor, Schering‐Plough, and Wyeth had no role in the study design or in the collection, analysis, or interpretation of the data, the writing of the manuscript, or the decision to submit the manuscript for publication. Publication of this article was not contingent upon approval by Abbott/AbbVie, Amgen, Centocor, Schering‐Plough, and Wyeth.

## Supporting information


**Disclosure form**.

**Supplementary Figure 1:** Directed acyclic graph (DAG) for the models to investigate the effect of NSAID Intake on progression in mSASSS.
