# Supplementary material for: The Impact of Nonsteroidal Anti‐Inflammatory Drugs on Radiographic Spinal Progression in Patients With Axial Spondyloarthritis: 10‐Year Results From an Inception Cohort
Source: Arthritis Rheumatol. 2026 Jan 26;78(3):582–91. doi: 10.1002/art.43447 (PMC12991912; doi:10.1002/art.43447)
Supplement: Supplementary file 2 — Supplementary Figure 1: Directed acyclic graph (DAG) for the models to investigate the effect of NSAID Intake on progression in mSASSS. [file ART-78-582-s002.docx]

**Supplementary material: The impact of non-steroidal anti-inflammatory drugs on radiographic spinal progression in patients with axial spondyloarthritis: 10-year results from an inception cohort**

**Supplementary Figure 1. Directed acyclic graph (DAG) for the models to investigate the effect of NSAID Intake on progression in mSASSS.**


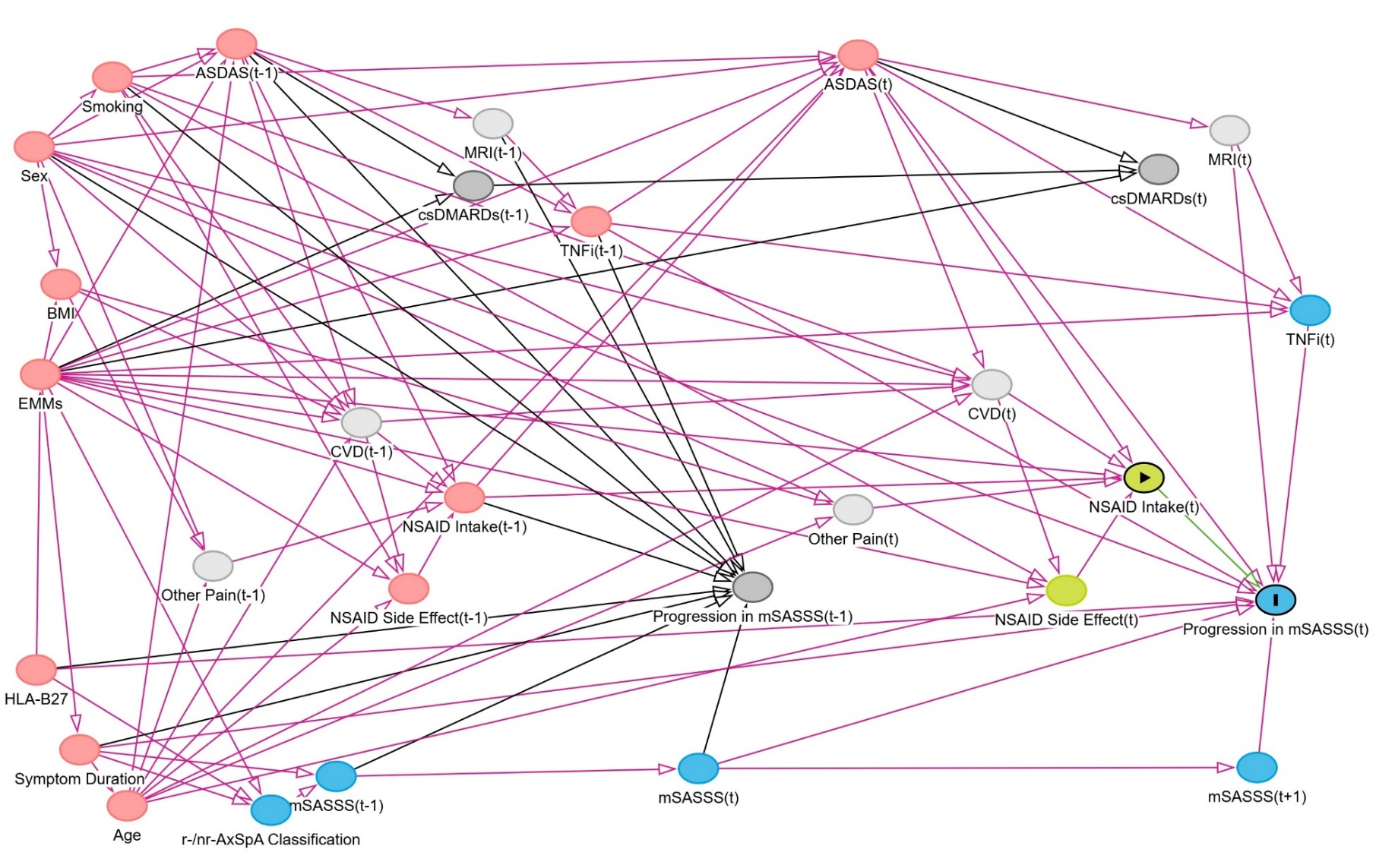


A Directed acyclic graph (DAG) is a graphical tool used to represent causal relationships between variables to ensure that confounding is properly accounted for in observational studies. The nodes (circles) represent variables and the directed edges (arrows) indicate assumed causal relationships. DAGs follow a strictly acyclic structure, meaning that there are no feedback loops or cycles.

Green node (Exposure): NSAID intake at time "t" is the primary exposure variable of interest.

Blue node (Outcome): Progression in mSASSS at time "t" is the dependent variable.

Light yellow nodes-Ancestors of Exposure: These variables affect the exposure (NSAID intake) but not the outcome (progression in mSASSS).

Light blue nodes-Ancestors of Outcome: These variables affect the outcome but not the exposure.

Pink nodes - ancestors of both exposure and outcome: These variables influence both exposure and outcome, and represent potential confounders that need to be accounted for.

Grey nodes - unobserved latent variables: These variables cannot be measured directly, but are assumed to exist based on prior knowledge.

Light grey nodes - other variables: These variables are included for completeness but are not directly involved in confounding or causal estimation.

**Paths in the DAG:**

Green line (Causal Path): The direct pathway from NSAID intake to progression in mSASSS, representing the effect of interest.

Pink lines (Biasing Paths): These represent non-causal pathways (backdoor paths) that introduce confounding. To obtain an unbiased estimate of the effect of NSAID intake, these paths must be blocked through proper adjustments.

Black lines (Neutral Paths): These connections do not introduce confounding or contribute directly to the causal effect.

**Minimal Sufficient Adjustment Sets:**

To estimate the total effect of NSAID Intake (t) on Progression in mSASSS (t), the following minimal sufficient adjustment sets were identified using DAGitty:

- ASDAS(t), ASDAS(t-1), Age, EMMs, NSAID Intake(t-1), Sex, Smoking
- ASDAS(t), Age, EMMs, Sex, TNFi(t-1)
- ASDAS(t), EMMs, Sex, Symptom Duration, TNFi(t-1)

These sets represent different combinations of confounders that, when adjusted for, allow an unbiased estimation of the causal effect of NSAID intake on progression in mSASSS.

This DAG explicitly models the time course of variables, with "t-1" representing the previous time point and "t" the current one. The figure was created using DAGitty v3.1 ([www.daggity.net](http://www.daggity.net)).

ASDAS, Axial Spondyloarthritis Disease Activity Score; BMI, Body mass index; csDMARDs, conventional synthetic disease-modifying antirheumatic drugs; CVD, Cardiovascular Disease; EMMs, extra musculoskeletal manifestations; HLA-B27, human leucocyte antigen B27; MRI, Magnetic resonance Imaging; mSASSS, modified Stroke Ankylosing Spondylitis Spine Score; NSAIDs, non-steroidal anti-inflammatory drugs; TNFi, tumour necrosis factor alpha inhibitor.
